# Supplementary material for: Velocities of transmission eigenchannels and diffusion
Source: Nat Commun. 2024 Mar 23;15:2606. doi: 10.1038/s41467-024-46748-0 (PMC10960809; doi:10.1038/s41467-024-46748-0)
Supplement: Supplementary file 1 — Supplementary Information [file 41467_2024_46748_MOESM1_ESM.pdf]

Supplementary Information for "Velocities of transmission eigenchannels and diffusion"
Genack *et al.*

Supplementary Information for "Velocities of transmission eigenchannels and diffusion"

Azriel Z. Genack[1,2*], Yiming Huang[1,2,3], Asher Maor[1,2,4] and Zhou Shi[1,2,5]
[1]*Department of Physics, Queens College of the City University of New York, Flushing, New York 11367, USA*
[2]*Physics Program, The Graduate Center of the City University of New York, New York New York, 10016, USA*
[3]*Jinhua No.1 High School, Zhejiang, 321000, China*
[4]*Kent Optronics, Inc., Hopewell Junction, New York 12533, USA*
[5]*OFS Labs, 19 School House Road, Somerset, New Jersey 08873, USA*

Contents

Supplementary Figures
1. Microwave measurements and experimental setup
2. Model of random sample
3. Scaling of eigenchannel velocities for $N = 64$
4. Determination of the scattering mean free path, ℓ_s
5. Simulation of pulsed transmission
6. Scaling of diffusion coefficient and its factors
7. Departure from linearity of $u(z)$ near the localization threshold

Supplementary Notes
1. Recursive Green's function simulations
2. Scaling of eigenchannel velocities for $N = 64$
3. Comparison of microwave and optical measurements of transmission.
4. Relationship between incident and reflected transmission eigenchannels
5. Energy density at the input surface
6. Normalization of energy density profile by its spatial average
7. Expression for nonlocal diffusion coefficient
8. Determining the scattering mean free path
9. Pulse propagation
10. Scaling of diffusion coefficient and its factors
11. Breakdown of diffusion in energy density profile

Supplementary References

Supplementary Figures

1. Microwave measurements and setup

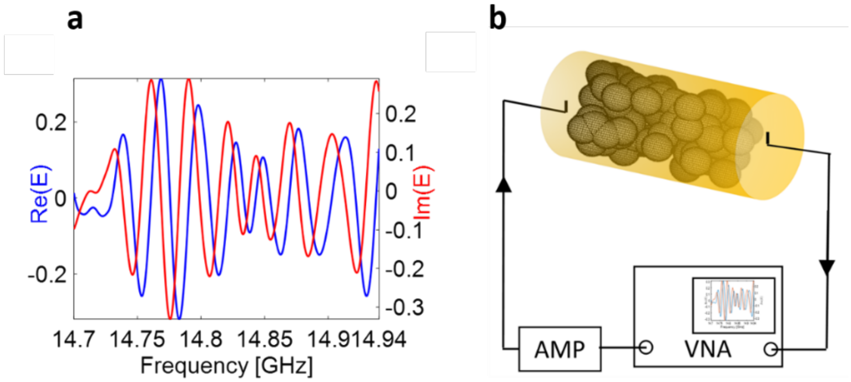

Supplementary Fig. 1 | Microwave measurements and setup. (a) Microwave spectra of in- and out-of-phase spectra of field transmission coefficients between source and receiver antennas on opposite sides of the sample for a single polarization pair on the incident and output surfaces of the sample. **b**, Schematic of experimental setup showing the vector network analyzer, amplifier of the source, and bent antennas.

2. Model of random sample

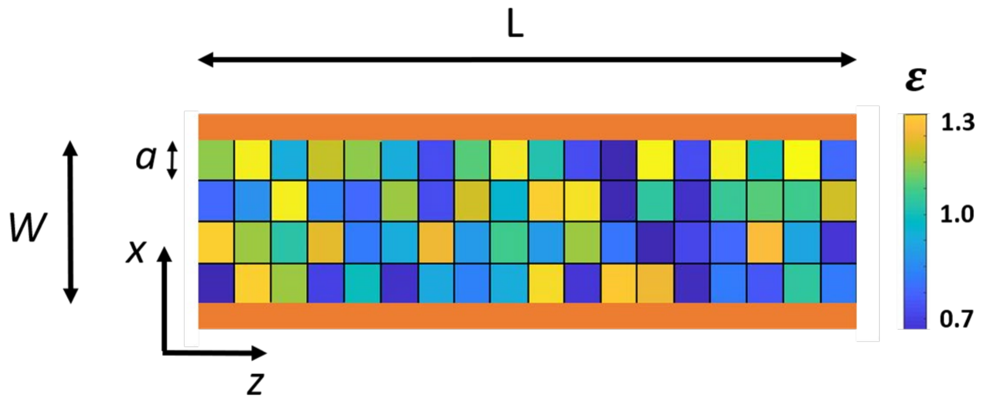

Supplementary Fig. 2 | Model of random sample. Model of random sample. The random sample is modelled by a lattice of square elements with sides of length $a = \lambda_0/2\pi$ and random values of the dielectric constant.

3. Scaling of eigenchannel velocities for $N = 64$

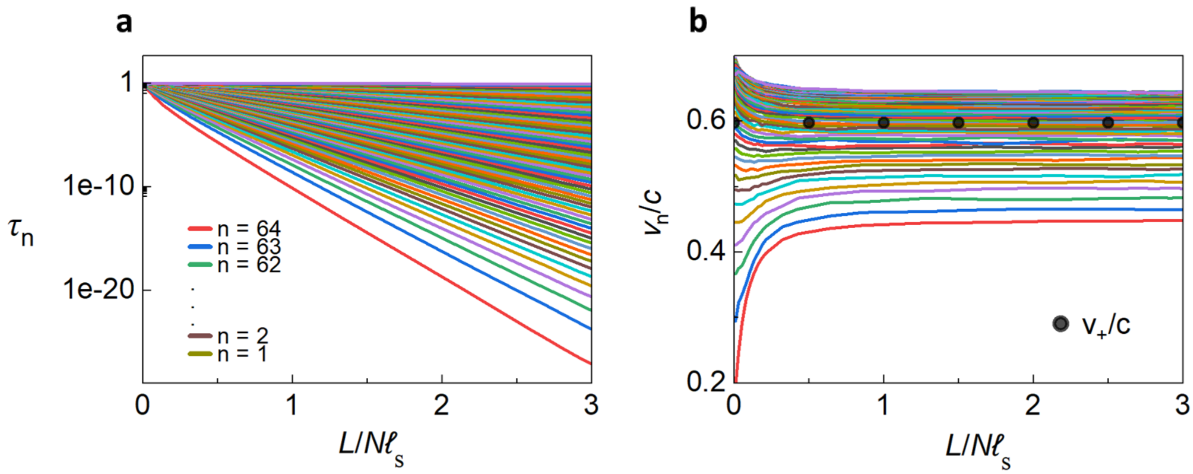

Supplementary Fig. 3 | Scaling of eigenchannel velocities for $N = 64$. (a) The transmission eigenvalues fall exponentially with sample length, while (b) the EVs approach distinct values asymptotically on length scales of a few times ℓ_s. For large N, this occurs for lengths shorter than the localization length of approximately $N\ell_s$.

4. Determination of the scattering mean free path, ℓ_s

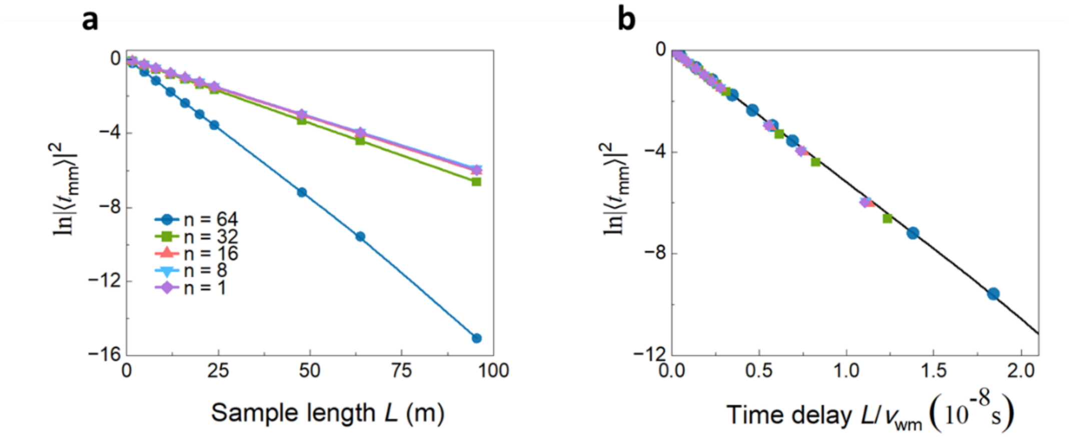

Supplementary Fig. 4 | Determination of the scattering mean free path, ℓ_s. (a) Scaling of the coherent flux of waveguide modes, $|\langle t_{mm}\rangle|^2$ in a sample with $N = 64$. (b) The decay of $|\langle t_{mm}\rangle|^2$ with coherent time delay $L/\mathrm{v}_{\mathrm{w}m}$, where $\mathrm{v}_{\mathrm{w}m}$ is the group delay of the mth waveguide mode. The decay time is the same for all waveguide modes.

5. Simulation of pulsed transmission

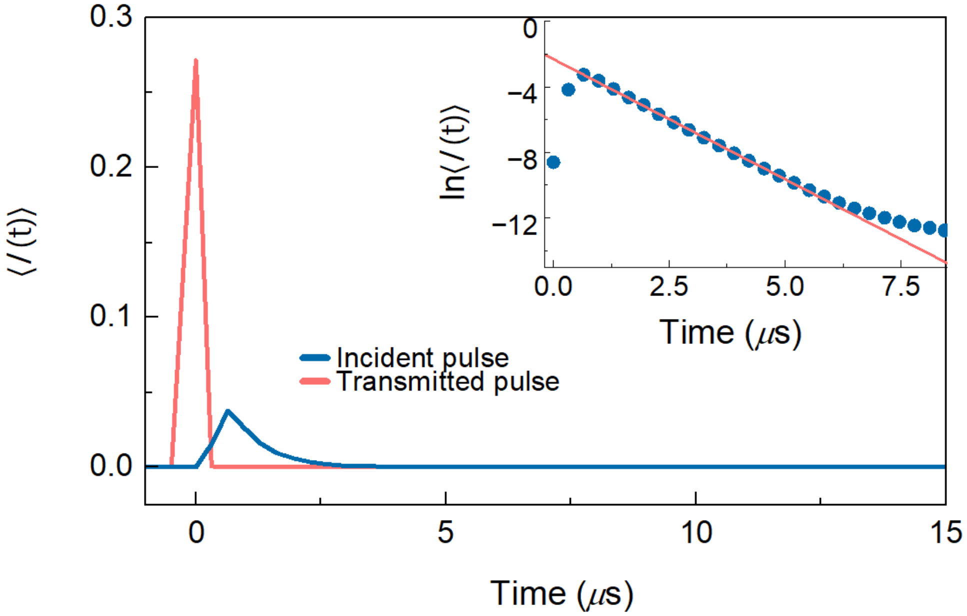

Supplementary Fig. 5 | Simulation of pulsed transmission. Pulsed transmission in sample with $N = 64$, $L = 7\ell_s$, and $\Delta\varepsilon = 0.3$. The inset shows that the decay of $\ln\langle I(t)\rangle$, with a rate that is proportional to the diffusion coefficient.

6. Scaling of diffusion coefficient and its factors

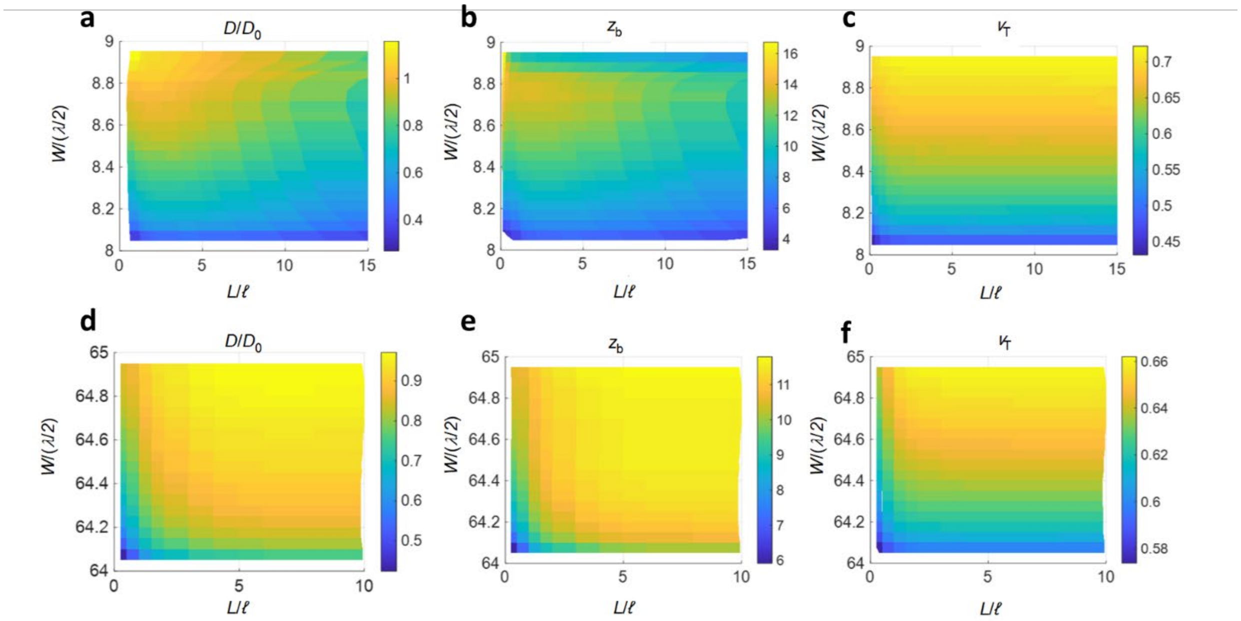

Supplementary Fig. 6 | Scaling of normalized diffusion coefficient and its factors. Scaling of $D/\widetilde{D_0}$, z_b and v_T with width and length are shown in the top row (a-c) for $N = 8$ and in the bottom row (d-f) for $N = 64$.

7. Departure from linearity of $u(z)$ near the localization threshold

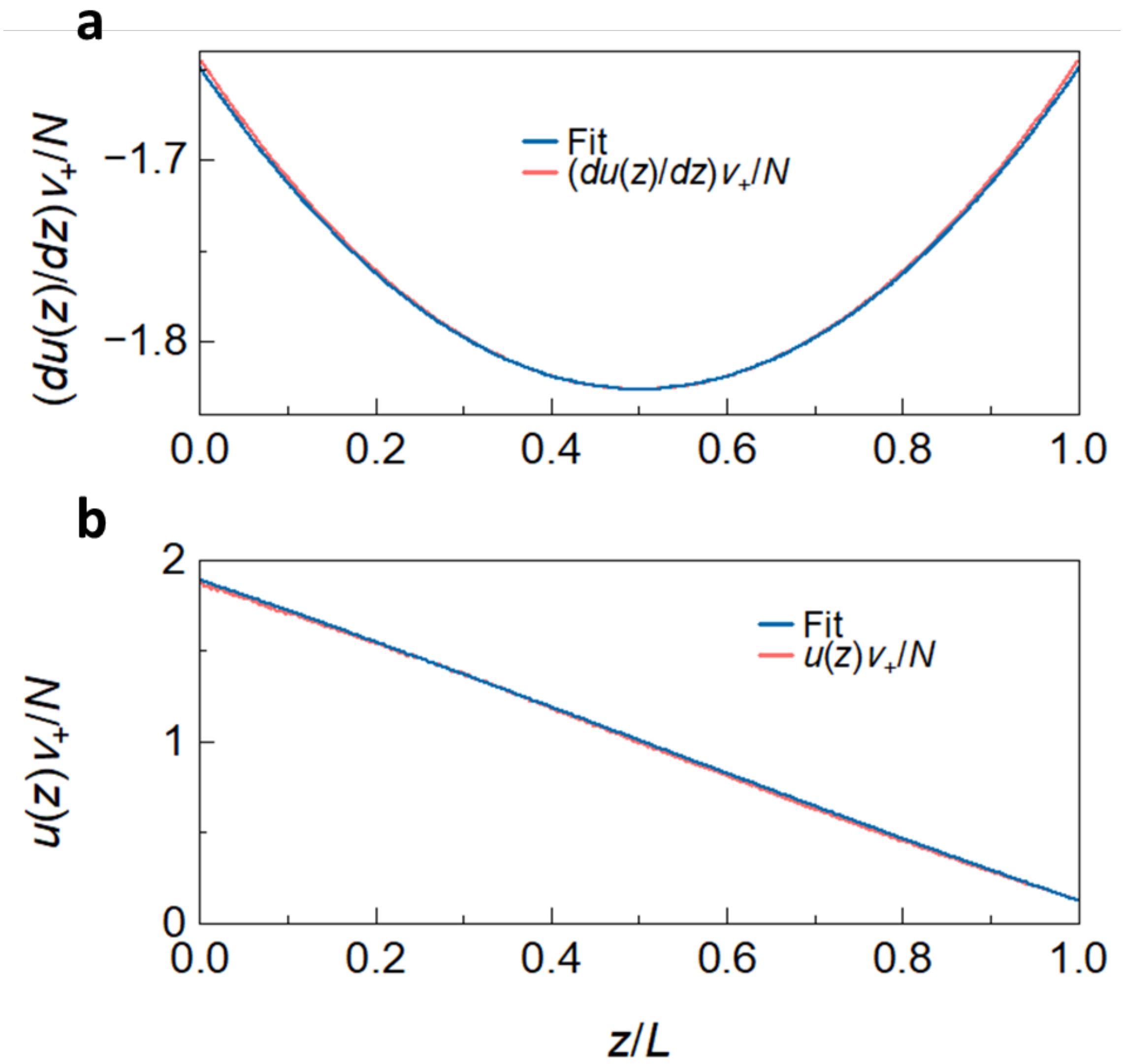

Supplementary Fig. 7 | Departure from linearity of $u(z)$ near the localization threshold. (a) The magnitude of the gradient of the energy density is greatest at the center of the sample. Since the position-dependent diffusion coefficient and the derivative of the energy density depend upon the distance from the nearest boundary, $(du(z)/dz)\frac{v_+}{N}$ is symmetric about the center of the sample. Further, since the deviation from linearity of the energy density is small, as seen I (b), we include only the leading order, quadratic, correction to the constant gradient of intensity within the sample. This gives an excellent fit. The parameters of the fit for this sample with $g = 1.078$ are given in Supplementry Note 11. (b) The energy density within the sample is seen to fall nearly linearly with depth for samples with length $g{\sim}1$.

Supplementary Notes

1. Recursive Green's function simulations

Simulations of electromagnetic wave propagation are carried out on model rectangular samples shown schematically in Supplementary Fig. 2. Samples of length L and width W are comprised of a lattice of squares with sides $a = \lambda/2\pi$ and random values of the dielectric constant, ε, drawn from a rectangular distribution $[1 - \Delta\varepsilon, 1 + \Delta\varepsilon]$ with $\Delta\varepsilon = 0.3$. The sample is open on the left and right and bounded on top and bottom by perfect reflectors. Thus, field excited from the open boundaries of the sample may be expressed in terms of the waveguide modes of the empty waveguide.

We employ the recursive Green's function method[3–5] to find the field in the kth column for a source on the left hand side of the sample. The sample is divided into K columns of width a, labeled 1, 2, …, k, … K. To find the field in each column of the sample, we first consider the field in an isolated column with random dielectric constant in each element within an otherwise empty waveguide. The field in the isolated k^{th} column is found by solving the homogeneous wave equation $(E_{kk} - H_{kk})\Psi = 0$, where Ψ is the electric field, $H_{kk} = \nabla^2 + k^2$, and E_{kk} is the eigenvalue of the wave equation. We refer to H_{kk} as a Hamiltonian because the wave equation is discretized in analogy to the tight-binding Hamiltonian used in electronic systems[3].

We seek to solve the equation $\left(E_{kk} - H_{kk}(\boldsymbol{r},\boldsymbol{r}')\right)G_{kk}(\boldsymbol{r},\boldsymbol{r}') = \delta(\mathbf{r} - \mathbf{r}')$ for a point source. We are interested in the causal solution for the Green's function, $G_{kk} = \lim_{\eta\to 0^+}(E_{kk} + i\eta - H_{kk})^{-1}$. The total Hamiltonian of the system is:

$$\begin{pmatrix} H_L & H_{1L} & 0 & 0 & 0 & 0 \\ H_{L1} & H_{11} & H_{12} & 0 & 0 & 0 \\ 0 & H_{21} & H_{22} & \ddots & 0 & 0 \\ 0 & 0 & \ddots & \ddots & H_{K-1,K} & 0 \\ 0 & 0 & 0 & H_{K,K-1} & H_{KK} & H_{KR} \\ 0 & 0 & 0 & 0 & H_{RK} & H_R \end{pmatrix}$$

Here, H_L is the Hamiltonian of the left lead, H_R is the Hamiltonian of the right lead, H_{kk} is the Hamiltonian of the kth column, and H_{kl} is the interaction term between the neighboring k^{th} and l^{th} columns. We begin with the surface Green's function of the left lead and proceed from left to right, connecting each column to the subsystem to its left.

The coupling between a kth column and the subsystem to its left is expressed via the Dyson equation, $G = G_0 + G_0VG$. G is the Green's functions of the connected columns from 1 to *k*. This is the matrix of solutions of the Hamiltonian submatrix from 1 to *k*. G_0 is the matrix of Green's functions of the connected columns from 1 to *k*-1 and the isolated column *k*, for a subsystem with the Hamiltonian

$$H_0 = \begin{pmatrix} H_L & H_{1L} & 0 & 0 & 0 \\ H_{L1} & H_{11} & H_{12} & 0 & 0 \\ 0 & H_{21} & H_{22} & \ddots & 0 \\ 0 & 0 & \ddots & \ddots & 0 \\ 0 & 0 & 0 & 0 & H_{kk} \end{pmatrix}$$

and V is the interaction terms between the two, given by

$$V = \begin{pmatrix} 0 & 0 & 0 & 0 & 0 \\ 0 & 0 & 0 & 0 & 0 \\ 0 & 0 & 0 & \ddots & 0 \\ 0 & 0 & \ddots & \ddots & H_{k-1,k} \\ 0 & 0 & 0 & H_{k,k-1} & 0 \end{pmatrix}$$

This gives the recursion equation

$$G^L_{k+1,1} = G^L_{k+1,k+1} V_{k+1,k} G^L_{k,1}, \qquad (10)$$

where the superscript L indicates the connected subsystem starting at the left side, G_{kl} is the Green's function of the connected subsystem at column k beginning from column l, and $G^L_{k+1,k+1} = [1 - G_{k+1,k+1}V_{k+1,k}G^L_{kk}V_{k,k+1}]^{-1}G_{k+1,k+1}$. A similar recursion relation is found by iterating from right to left. The left-to-right and right-to-left parts are then combined to obtain the result, $G_{k1} = [1 - G^L_{kk}V_{k,k+1}G^R_{k+1,k+1}V_{k+1,k}]^{-1}G^L_{k1}$, which allows us to calculate the Green's functions for all columns from 1 to K. The Green's functions are used to calculate the energy density, TM, transmission eigenvalues and EVs, and the energy density in each TE.

2. Scaling of eigenchannel velocities for $N = 64$

In contrast to the exponential decay of transmission eigenvalues with length in samples with $N = 8$, the EVs asymptotically approach a constant value with increasing length, as seen in Fig. 2(a,b). Most EVs approach an asymptotic value at lengths shorter than the localization length of approximately $N\ell_s$, but the EV of the transmission eigenchannels with the lowest transmission approaches its asymptotic value at a length which is comparable to $N\ell_s$. To check whether the approach of EVs to their asymptotic values is related to the onset of multiple scattering or of localization, we carried out simulations in samples with the same disorder of $\Delta\varepsilon = 0.3$ but with $N = 64$. The localization length is then much longer than the scattering mean free path. The results in Supplementary Fig. 3 show that the EV of low transmission eigenchannels close approach their asymptotic values for lengths decidedly shorter than $N\ell_s$. This indicates that the evolution of values of EVs with length is related to ℓ_s and not $N\ell_s$. Thus, the EV do not change in this sample in the crossover to Anderson localization and their distinct values are not related to Anderson localization.

3. Comparison of microwave and optical measurements of transmission

The nature of transmission and energy density within a random medium depends upon the excitation. When the TM can be measured, it is possible to excite the medium with the sum of all incident waveguide modes, as is the case in the present paper. This occurs naturally in measurements of electrical conductance. it is possible to excite Lower values of EVs for channels with smaller τ_n is consistent with measurements of a drop in transmission of a laser beam directed at a random dielectric slab with increasing angle of incidence, which corresponds to a reduced normal component of the velocity of the light [26]. The transmission through the slab is $T(\theta) = \frac{z_p \cos\theta' + z_b}{L + 2z_b}$, where z_p is the distance traveled by the beam before the direction light is randomized, and θ' is the angle of refraction within the sample.

The derivative of energy density is constant within a random medium and z_{b} is clearly defined when the flux is the same in all incident channels. More generally, as for the case of a laser beam incident on a random slab, the energy density will be a weighted superposition of the eigenchannels and will produce a different spatial profile of energy density, even deep within a random medium.

4. Relationship between incident and reflected transmission eigenchannels

For a dissipationless quasi-1D medium with reciprocity, the scattering matrix can be expressed as $S = \begin{bmatrix} r & t' \\ t & r' \end{bmatrix}$, where r and t are the reflection and transmission matrices for a wave incident from the left and the corresponding matrices for excitation from the right are primed.

Reciprocity implies $S^T = S$, while the conservation of flux in a lossless medium implies $S^{\dagger}S = 1$. The polar decomposition of the scattering matrix [,] gives

$$S = \begin{bmatrix} V_s & 0 \\ 0 & U_s \end{bmatrix} \begin{bmatrix} -\sqrt{1-\tau} & \sqrt{\tau} \\ \sqrt{\tau} & \sqrt{1-\tau} \end{bmatrix} \begin{bmatrix} V_s{}^T & 0 \\ 0 & U_s{}^T \end{bmatrix} = \begin{bmatrix} -V_s\sqrt{1-\tau}V_s{}^T & V_s\sqrt{\tau}U_s{}^T \\ U_s\sqrt{\tau}V_s{}^T & U_s\sqrt{1-\tau}U_s{}^T \end{bmatrix}, \quad (11)$$

where V_s and U_s are unitary matrices. Here,

$$t = U_t\sqrt{\tau}V_t^{\dagger} = U_s\sqrt{\tau}V_s{}^T = U_s\sqrt{\tau}(V_s{}^*)^{\dagger}, \text{ so } U_t = U_s, V_t = V_s{}^* \quad (12a)$$

$$t' = U_{t'}\sqrt{\tau}V_{t'}^{\dagger} = V_s\sqrt{\tau}U_s{}^T = V_s\sqrt{\tau}(U_s{}^*)^{\dagger}, \text{ so } U_{t'} = V_s, V_{t'} = U_s{}^* \quad (12b)$$

$$r = U_r\sqrt{\tau}V_r^{\dagger} = -V_s\sqrt{1-\tau}V_s{}^T = -V_s\sqrt{1-\tau}(V_s{}^*)^{\dagger}, \text{ so } U_r = -V_s, V_r = V_s{}^* \quad (12c)$$

$$r' = U_{r'}\sqrt{1-\tau}V_{r'}^{\dagger} = U_s\sqrt{1-\tau}U_s{}^T = U_s\sqrt{1-\tau}(U_s{}^*)^{\dagger}, \text{ so } U_{r'} = U_s, V_{r'} = U_s{}^* \quad (12d)$$

With these relations, and the knowledge of τ, U_t and V_t, we can obtain t', t, r' and, as thus the full scattering matrix. In the main text, which deals primarily with the TM, we have indicated U_t by U and V_t by V. Thus, for the transmission matrix, the incident singular vector is V_t, and the transmitted singular vector is U_t. For the reflection matrix, the incident eigenvector is V_t, the reflected singular vector is $-V_t^*$.

The velocity of the nth transmitted eigenchannel is $\mathrm{v}_{n,t} = \sum_{m=1}^{N} v_m \left|V_{t,n,m}\right|^2$, the velocity of the nth reflected eigenchannel is $\mathrm{v}_{n,r} = \sum_{m=1}^{N} \mathrm{v}_m \left|-V_{t,n,m}^*\right|^2 = \mathrm{v}_{n,t}$.

5. Energy density at the input surface

Here we show that the average energy density at the input surface is equal to the sum of averages of the energy densities of the incident and reflected waves, $u_n(0) = u_{n,\mathrm{i}}(0) + u_{n,\mathrm{r}}(0)$. This is a consequence of the proportionality of the reflected transmission eigenchannel and the complex conjugate of the incident TE, which was shown in Appendix D. Since the demonstration is based on relationships in single configurations, we distinguish between a property of a single configuration and the average over configurations by using a bracket for the latter.

Since the reflected transmission eigenchannel is proportional to the complex conjugate of the incident TE, the longitudinal velocities of the incident and reflected transmission eigenchannels in each sample configuration are the same, $\mathrm{v}_{n,\mathrm{i}} = \mathrm{v}_{n,\mathrm{r}}$. The linear energy density in a transmission eigenchannel at the sample input for a single sample configuration normalized

to the spatial average of the energy density over all transmission eigenchannels can therefore be expressed as an amplitude squared of a sum of waves traveling to the right, $A_n(0)$, and left,

$$B_n(0), u_n(0)\mathrm{v}_+ = |A_n(0) + B_n(0)|^2, \quad (13)$$

with $B_n(0) = r_n A_n^*(0)$. Here r_n is the field reflection coefficient for the nth transmission eigenchannel in a single configuration, with $r_n^2 = 1 - \tau_n$. We can, thus, write

$$A_n(0) + B_n(0) = A_n(0) + r_n A_n^*(0). \quad (14)$$

Writing $A_n(0) = r_n A_n(0) + (1 - r_n)A_n(0)$, leads to
$A_n(0) + B_n(0) = r_n\big(A_n(0) + A_n^*(0)\big) + (1 - r_n)A_n(0) = 2r_n Re\big(A_n(0)\big) + (1 - r_n)A_n(0)$.

Averaging $u_n(0)\mathrm{v}_+ = \big(A_n(0) + B_n(0)\big)\big(A_n^*(0) + B_n^*(0)\big)$ over a random ensemble gives,
$\langle u_n(0)\rangle \mathrm{v}_+ = \langle 4r_n^2\big[Re\big(A_n(0)\big)\big]^2 - (1 - r_n)^2|A_n(0)|^2 + 4r_n\big[Re\big(A_n(0)\big)\big]^2(1 - r_n)\rangle$.
Since, $\langle Re\big(A_n(0)\big)^2\rangle = \langle Im\big(A_n(0)\big)^2\rangle$, $\langle |A_n(0)|^2\rangle = 2\,\langle Re\big(A_n(0)\big)^2\rangle$. This gives,
$\langle u_n(0)\rangle \mathrm{v}_+ = \langle \big[Re\big(A_n(0)\big)\big]^2 4r_n^2 - \big[Re\big(A_n(0)\big)\big]^2 2(1 - r_n)^2 + \big[Re\big(A_n(0)\big)\big]^2 4r_n(1 - r_n)\rangle$.
Thus,
$\langle u_n(0)\rangle \mathrm{v}_+ = \langle \big[Re\big(A_n(0)\big)\big]^2[4r_n^2 - 2(1 - r_n)^2 + 4r_n(1 - r_n)]\rangle = \langle \big[Re\big(A_n(0)\big)\big]^2[2(1 + r_n^2)]\rangle$.
But, $\langle 2(1 + r_n^2)\rangle = 2\langle(1 + (1 - \tau_n)\rangle = 2\langle 2 - \tau_n\rangle$. Hence,
$\langle u_n(0)\rangle \mathrm{v}_+ = \langle 2[Re((1 - \tau_n))]^2(2 - \tau_n)\rangle = \langle |A_n(0)|^2(1 + (1 - \tau_n))\rangle$.
Finally, $\langle u_n(0)\rangle \mathrm{v}_+ = \langle u_{n,\mathrm{i}}(0)\rangle \mathrm{v}_+ + \langle u_{n,\mathrm{r}}(0)\rangle \mathrm{v}_+$, so that

$$\langle u_n(0)\rangle = \langle u_{n,\mathrm{i}}(0)\rangle + \langle u_{n,\mathrm{r}}(0)\rangle. \quad (15)$$

6. Normalization of energy density profile by its spatial average

Energy density profiles for ensembles with different values of g are shown in Fig. 6(a). The energy density profile $u(z)$ is normalized by its average over configuration and position. This average is obtained by noting that the average energy densities excited in each transmission eigenchannel on the opposite side of the sample for excitation from the left, $u_n(L)$, and the right, $u_n'(0)$, are equal, $u_n'(0) = u_n(L) = \frac{\tau_n}{\mathrm{v}_n}$. Thus, the sum of energy density at the input for excitation in all channels on both sides of the sample is $U(0) = \sum_{n=1}^{N}[u_n(0) + u_n'(0)] = \sum_{n=1}^{N} u(0) + u(L)$. Using Eq. (2), gives, $U(0) = \frac{2N}{\mathrm{v}_+}$. Together with the result above that $\rho_{\omega,L} = \frac{N}{\pi \mathrm{v}_+}$, this gives $U(0) = \pi\rho_{\omega,L}/2$[6,7]. Since the spatial average of the energy density excited by all channels from either the left or right are equal,

$$\langle u(z)\rangle_z = \langle u'(z)\rangle_z = \frac{1}{2}U(z) = \frac{N}{\mathrm{v}_+}. \quad (16)$$

7. Expression for nonlocal diffusion coefficient

Substituting the expression for g in Eq. (6), $g = \frac{2Nz_b}{L+2z_b}\frac{\mathrm{v_T}}{\mathrm{v}_+}$, into the expression for D in Eq. (4b) gives

$$D = \frac{\frac{gL}{2}}{\frac{N}{\mathrm{v}_+} - \frac{g}{\mathrm{v_T}}} = \frac{\frac{2Nz_b\,\mathrm{v_T}L}{L+2z_b\mathrm{v}_+2}}{\frac{N}{\mathrm{v}_+} - \frac{\frac{2Nz_b\,\mathrm{v_T}}{L+2z_b\mathrm{v}_+}}{\mathrm{v_T}}}. \quad (17)$$

Straightforward manipulation gives,

$$D = \frac{\frac{z_b}{L+2z_b}\frac{\mathrm{v_T}}{\mathrm{v}_+}L}{\frac{1}{\mathrm{v}_+} - \frac{\frac{2z_b}{L+2z_b}\frac{\mathrm{v_T}}{\mathrm{v}_+}}{\mathrm{v_T}}} = \frac{\frac{z_b}{L+2z_b}\frac{\mathrm{v_T}}{\mathrm{v}_+}L}{\frac{\mathrm{v_T}}{\mathrm{v}_+} - \frac{2z_b}{L+2z_b}\frac{\mathrm{v_T}}{\mathrm{v}_+}}\mathrm{v_T}$$

$$= \frac{\frac{z_b}{L+2z_b}L}{1 - \frac{2z_b}{L+2z_b}}\mathrm{v_T} = \frac{z_b L}{L+2z_b-2z_b}\mathrm{v_T} = z_b\mathrm{v_T},$$

which is the result of Eq. (7).

8. Determining the scattering mean free path

Since the scale of disorder in the scattering model considered in simulations is much smaller than the wavelength, $a = \lambda/2\pi$, scattering is nearly isotropic, and resonances do not affect the energy velocity. We therefore expect the scattering mean free time, τ_{s}, to be nearly independent of the direction of propagation of the wave. This is confirmed in the analysis below, which shows that the scattering mean free times for different incident waveguide modes, which have different transverse velocities, are identical.

The scattering mean free path ℓ_s in a sample with $\Delta\varepsilon = 0.3$, $N = 64$ and width $W = 64.5\frac{\lambda_0}{2}$ is found from the decay of the coherent flux vs. ballistic time delay of the waveguide modes. The scaling of the coherent flux of waveguide modes, $|\langle t_{mm}(L)\rangle|^2$, is shown in Supplementary Fig. 4(a). $|\langle t_{mm}(L)\rangle|^2$ is the ensemble average of the flux of the mth waveguide mode at the output of a random sample of length L. The waveguide modes with higher index, m, which have smaller group velocities, $\mathrm{v}_{\mathrm{w}m}$, decay more rapidly.
The decay of $|\langle t_{mm}\rangle|^2$ vs. coherent time delay $L/\mathrm{v}_{\mathrm{w}m}$, is shown in Supplementary Fig. 4(b). The decay times of the coherent flux for the mth waveguide mode is[8], $\tau_{s,m} = \frac{\ell_{s,m}}{\mathrm{v}_m}$. Since all the plots collapse to a single curve and fall exponentially to give a single mean free scattering time τ_{s}, and the speed of the wave in the medium is c, the scattering mean free path is $\ell_s = c\tau_{\mathrm{s}} = 16.0$ m.

9. Pulse propagation

Under steady-state illumination, $D(W,L)$ can be expressed via Fick's first law in terms of the parameters of the TEs, τ_n and v_n, as in Eq. (4a). In the time domain, $D(W,L)$ is given by Fick's second law as the ratio of the partial derivative of concentration with time and the Laplacian of the concentration. In samples thicker than five times the mean free path, the transmitted intensity in a diffusive medium falls exponentially in time once higher order diffusion modes have decayed, with a decay rate $1/\tau_{\mathrm{D}} = \pi^2 D/(L+2z_{\mathrm{b}})^2$ [9–11,10]. This is the decay rate of the quasi-normal modes or resonances of the medium and equals the linewidth of quasi-normal modes [12,13].

The transmission due to an incident Gaussian pulse is obtained by multiplying field transmission spectra with the Gaussian spectrum of the envelope for the field pulse and Fourier

transforming into the time domain. The ensemble average of the transmitted intensity for the sample for which steady-state results are shown in Fig. 4(a) with $N = 64$ and $L = 7\ell_s$, at which $\frac{D}{\widetilde{D_0}}$ reaches its peak value of 0.91, is shown in Fig. 4(a). The transmitted pulse decays exponential with decay time $\tau_{\mathrm{D}} = 0.68$ μs $\pm$ 0.01. Beyond a delay of 5 μs, the decay rate slows due to the increasing relative weight of longer lived quasi-normal modes[12–14]. The diffusion coefficient given by $D = \frac{(L+2z_{\mathrm{b}})^2}{\pi^2\tau_{\mathrm{D}}}$[11], which is equal to $2.16 \times 10^9 \pm 0.02\ \mathrm{m}^2/\mathrm{s}$, obtained with z_{b} taken as its average value over the spectrum for the incident Gaussian pulse, $z_{\mathrm{b}} = 11.4$. The average scattering mean free path over the spectrum of the incident pulse of $\ell_s = 16.0 \ \pm 0.15$ m, gives $\widetilde{D_0} = \frac{1}{2}c\ell_s = 2.40 \times 10^9$ m^2/s and $D/\widetilde{D_0} = 0.90 \pm 0.015$, in agreement with the state-diffusion coefficient for this sample, as seen in Fig. 4(a). The uncertainty in $D/\widetilde{D_0}$ is due to different values of the slope of the curve in the inset obtained for different time ranges.

Though the diffusion coefficients found in steady-state and in time-domain simulations agree, still D falls 10% below $\widetilde{D_0}$. Several factors contribute to this difference. At the length at which $D(L)$ reaches its peak value, the diffusion coefficient is already lowered below the bare diffusion coefficient due to weak localization[12,13,15]. The bare diffusion coefficient might be obtained by extrapolating the linear region of $D(L)/\widetilde{D_0}$ in Fig. 4(a) back to $L = 0$. This gives, $D(0)/\widetilde{D_0} = 0.95$. In the diffusive regime, the length dependent diffusion coefficient, $D(L)$, falls linearly with length from the bare diffusion coefficient with the fractional reduction in $D(L)$ proportional to $\frac{1}{g_{Th}} \sim \tau_{Th}/\tau_{\mathrm{T}}$ because of the increasing probability of a path looping back upon a coherence length of the path with increasing sample thickness [10]. Similarly, the time dependent diffusion coefficient proportional to the decay rate of $\ln\langle I(t)\rangle$ falls linearly with delay time, with the drop in decay rate proportional to t/τ_{T}[12,13].

Another reason that $D/\widetilde{D_0}$ may fall below unity is that $\widetilde{D_0} = \frac{1}{2}c\ell_s$ may be larger than the bare diffusion coefficient $D_0 = \frac{1}{2}\mathrm{v_E}\ell$[16]. There are two countervailing factors, ℓ may be larger than ℓ_s, while $\mathrm{v_E}$ may be smaller than c. These corrections are likely to be small since $a = \lambda_0/2\pi$, so that the individual scattering elements are too small to support resonances so that $\mathrm{v_E} \sim c$, and scattering might be nearly isotropic with $\ell \sim \ell_s$. However, it should be noted that the prevailing assumption that scattering is isotropic and is independent of sample dimensions, which leads to the classical result for the diffusion coefficient, is shown here not to hold for wave diffusion in bounded media.

10. Scaling of diffusion coefficient and its factors

The scale-dependent diffusion coefficient $D(W, L)$ relative to $\widetilde{D_0} = \frac{1}{2}c\ell_s$ and the factors of the diffusion coefficient $z_{\mathrm{b}}(W, L)$ and $\mathrm{v_T}(W, L)$, as given in Eq. (7), are given in Supplementary Fig. 6. Results for samples with disorder $\Delta\varepsilon = 0.3$ and for $N = 8$ and $N = 64$ are shown, respectively, in the top and bottom rows over the full range of W for each N and for a wide range of L/ℓ_s. The variation with width is smaller for larger N, but is still substantial for $N = 64$.

11. Breakdown of diffusion in energy density profile

We find that energy density within the sample falls linearly with depth for sample with lengths nearly equal to the localization length $L = \xi$ and $g = 1$. Here we consider the variation of energy density in an ensemble of samples with $g = 1.078$. The sample width is $W = (N + \frac{1}{2})\frac{\lambda_0}{2} =$

$8.5\frac{\lambda_0}{2}$. The derivative of the normalized energy density inside the medium is shown as the blue curve in Supplementary Fig. 7(a). Since the position-dependent diffusion coefficient[1,2] and the derivative of the energy density depend upon the distance from the nearest boundary, $(du(z)/dz)\frac{\mathrm{v}_+}{N}$ is symmetric about the center of the sample. To second order in the displacement from the center of the sample, $(du(z)/dz)\frac{\mathrm{v}_+}{N} = a + b\left[(z - \frac{L}{2})/(L/2)\right]^2$, where $a = \frac{d\langle u(z)\rangle}{dz}|_{z=L/2}\frac{\mathrm{v}_+}{N}$ and $b = \frac{d^2\langle u(z)\rangle}{dz^2}|_{z=\frac{L}{2}}\frac{\mathrm{v}_+}{N}$. The fit of this function to $(du(z)/dz)\frac{\mathrm{v}_+}{N}$ gives $a = -1.826$, and $b = 0.177$, and is displayed as the red curve. $u(z)\frac{\mathrm{v}_+}{N}$ found in simulations (blue curve) and computed with use of the parameters a and b found in (a) (red curve) are seen to overlap in Supplementary Fig. 7(b).

Supplementary References

1. Baranger, H. U., DiVincenzo, D. P., Jalabert, R. A. & Stone, A. D. Classical and quantum ballistic-transport anomalies in microjunctions. *Phys. Rev. B Condens. Matter* **44**, 10637–10675 (1991).
2. Wimmer, M. Quantum transport in nanostructures: From computational concepts to spintronics in graphene and magnetic tunnel junctions. (Doctoral Dissertation, 2009).
3. G. Metalidis. Electronic Transport in Mesoscopic Systems. (Doctoral Dissertation, 2007).
4. Fyodorov, Y. V. & Sommers, H.-J. Statistics of resonance poles, phase shifts and time delays in quantum chaotic scattering: Random matrix approach for systems with broken time-reversal invariance. *J. Math. Phys.* **38**, 1918–1981 (1997).
5. Huang, Y., Kang, Y. & Genack, A. Z. Wave excitation and dynamics in non-Hermitian disordered systems. *Phys. Rev. Res.* **4**, 013102 (2022).
6. Shi, Z. & Genack, A. Z. Diffusion in translucent media. *Nat. Commun.* **9**, 1862 (2018).
7. Genack, A. Z. & Drake, J. M. Relationship between optical intensity, fluctuations and pulse propagation in random media. *Europhys. Lett.* **11**, 331 (1990).
8. Yoo, K. M., Liu, F. & Alfano, R. R. When does the diffusion approximation fail to describe photon transport in random media? *Phys. Rev. Lett.* **64**, 2647–2650 (1990).

9. Johnson, P. M., Imhof, A., Bret, B. P. J., Rivas, J. G. & Lagendijk, A. Time-resolved pulse propagation in a strongly scattering material. *Phys. Rev. E* **68**, 016604 (2003).

10. Chabanov, A. A., Zhang, Z. Q. & Genack, A. Z. Breakdown of Diffusion in Dynamics of Extended Waves in Mesoscopic Media. *Phys. Rev. Lett.* **90**, 203903 (2003).

11. Mirlin, A. D. Statistics of energy levels and eigenfunctions in disordered systems. *Phys. Rep.* **326**, 259–382 (2000).

12. Wang, J. & Genack, A. Z. Transport through modes in random media. *Nature* **471**, 345–348 (2011).

13. Altshuler, B. L., Khmel'nitzkii, D., Larkin, A. I. & Lee, P. A. Magnetoresistance and Hall effect in a disordered two-dimensional electron gas. *Phys. Rev. B* **22**, 5142–5153 (1980).

14. van Albada, M. P., van Tiggelen, B. A., Lagendijk, A. & Tip, A. Speed of propagation of classical waves in strongly scattering media. *Phys. Rev. Lett.* **66**, 3132–3135 (1991).
